# Stress-Mediated Increases in Systemic and Local Epinephrine Impair Skin Wound Healing: Potential New Indication for Beta Blockers

**DOI:** 10.1371/journal.pmed.1000012

**Published:** 2009-01-13

**Authors:** Raja K Sivamani, Christine E Pullar, Catherine G Manabat-Hidalgo, David M Rocke, Richard C Carlsen, David G Greenhalgh, R. Rivkah Isseroff

**Affiliations:** 1 Department of Dermatology, University of California, Davis, School of Medicine, Davis, California, United States of America; 2 Division of Biostatistics, University of California, Davis, School of Medicine, Davis, California, United States of America; 3 Department of Physiology and Membrane Biology, University of California, Davis, School of Medicine, Davis, California, United States of America; 4 Department of Surgery, University of California, Davis, School of Medicine, Davis, California, United States of America; 5 Shriners Hospitals for Children Northern California, Sacramento, California, United States of America; 6 Wound Service, Department of Veterans Affairs, Northern California Health Care System, Mather, California, United States of America; Vanderbilt, United States of America

## Abstract

**Background:**

Stress, both acute and chronic, can impair cutaneous wound repair, which has previously been mechanistically ascribed to stress-induced elevations of cortisol. Here we aimed to examine an alternate explanation that the stress-induced hormone epinephrine directly impairs keratinocyte motility and wound re-epithelialization. Burn wounds are examined as a prototype of a high-stress, high-epinephrine, wound environment. Because keratinocytes express the β2-adrenergic receptor (β2AR), another study objective was to determine whether β2AR antagonists could block epinephrine effects on healing and improve wound repair.

**Methods and Findings:**

Migratory rates of normal human keratinocytes exposed to physiologically relevant levels of epinephrine were measured. To determine the role of the receptor, keratinocytes derived from animals in which the β2AR had been genetically deleted were similarly examined. The rate of healing of burn wounds generated in excised human skin in high and low epinephrine environments was measured. We utilized an in vivo burn wound model in animals with implanted pumps to deliver β2AR active drugs to study how these alter healing in vivo. Immunocytochemistry and immunoblotting were used to examine the up-regulation of catecholamine synthetic enzymes in burned tissue, and immunoassay for epinephrine determined the levels of this catecholamine in affected tissue and in the circulation. When epinephrine levels in the culture medium are elevated to the range found in burn-stressed animals, the migratory rate of both cultured human and murine keratinocytes is impaired (reduced by 76%, 95% confidence interval [CI] 56%–95% in humans, *p* < 0.001, and by 36%, 95% CI 24%–49% in mice, *p* = 0.001), and wound re-epithelialization in explanted burned human skin is delayed (by 23%, 95% CI 10%–36%, *p* = 0.001), as compared to cells or tissues incubated in medium without added epinephrine. This impairment is reversed by β2AR antagonists, is absent in murine keratinocytes that are genetically depleted of the β2AR, and is reproduced by incubation of keratinocytes with other β2AR-specific agonists. Activation of the β2AR in cultured keratinocytes signals the down-regulation of the AKT pathway, accompanied by a stabilization of the actin cytoskeleton and an increase in focal adhesion formation, resulting in a nonmigratory phenotype. Burn wound injury in excised human skin also rapidly up-regulates the intra-epithelial expression of the epinephrine synthesizing enzyme phenylethanolamine-N-methyltransferase, and tissue levels of epinephrine rise dramatically (15-fold) in the burn wounded tissue (values of epinephrine expressed as pg/ug protein ± standard error of the mean: unburned control, 0.6 ± 0.36; immediately postburn, 9.6 ± 1.58; 2 h postburn, 3.1 ± 1.08; 24 h post-burn, 6.7 ± 0.94). Finally, using an animal burn wound model (20% body surface in mice), we found that systemic treatment with βAR antagonists results in a significant increase (44%, 95% CI 27%–61%, *p* < 0.00000001) in the rate of burn wound re-epithelialization.

**Conclusions:**

This work demonstrates an alternate pathway by which stress can impair healing: by stress-induced elevation of epinephrine levels resulting in activation of the keratinocyte β2AR and the impairment of cell motility and wound re-epithelialization. Furthermore, since the burn wound locally generates epinephrine in response to wounding, epinephrine levels are locally, as well as systemically, elevated, and wound healing is impacted by these dual mechanisms. Treatment with beta adrenergic antagonists significantly improves the rate of burn wound re-epithelialization. This work suggests that specific β2AR antagonists may be apt, near-term translational therapeutic targets for enhancing burn wound healing, and may provide a novel, low-cost, safe approach to improving skin wound repair in the stressed individual.

## Abstract 2

Rivkah Isseroff and colleagues describe how stress-induced elevation of epinephrine levels can impair the healing of burns in mice and suggest that β2 adrenergic receptor antagonists may have a role in improving skin wound repair.

## Editors' Summary

**Background.:**

Skin—the largest organ in the human body—protects the rest of the body against infection by forming an impervious layer over the whole external body surface. Consequently, if this layer is damaged by rubbing, cutting, or burning, it must be quickly and efficiently repaired. Wound repair (healing) involves several different processes. First, the clotting cascade stops bleeding at the wound site and immune system cells attracted into the site remove any bacteria or debris in the wound. Various factors are released by the immune cells and the other cells in and near the damaged area that encourage the migration of several different sorts of cells into the wound. These cells proliferate and prepare the wound for “re-epithelialization.” In this process, keratinocytes (a type of epithelial cell that makes a tough, insoluble protein called keratin; epithelial cells cover all the surfaces of the body) migrate into the wound site and form a new, intact epithelial layer. If any of these processes fail, the result can be a chronic (long-lasting) nonhealing wound. In particular, if the wound does not re-epithelialize, it remains open and susceptible to infection and loss of body fluids.

**Why Was This Study Done?:**

One factor that impairs the repair of skin wounds is stress. In stressful situations (including situations in which wounds are likely to occur), the human body releases several chemicals that prepare the body for “fight or flight,” including cortisol and epinephrine (also called adrenaline). Most scientists ascribe the effects of stress on wound healing to stress-induced increases in cortisol, but might stress-induced epinephrine also affect wound healing? In this study, the researchers test whether epinephrine impairs keratinocyte migration and re-epithelialization of burn wounds (keratinocytes have a receptor for epinephrine called the β2 adrenergic receptor [β2AR] on their cell surface that allows them to respond to epinephrine). They chose to study burn wounds for two reasons. First, major burns cause a massive release of stress chemicals into the bloodstream that raises blood levels (systemic levels) of cortisol and epinephrine for days or weeks after the initial trauma. Second, despite recent therapeutic advances, many people still die from major burns (4,000 every year in the USA alone) so there is a pressing need for better ways to treat this type of wound.

**What Did the Researchers Do and Find?:**

The researchers investigated the effects of epinephrine on wound healing in three types of experiments. First, they looked at the effect of epinephrine on keratinocytes growing in dishes (in vitro experiments). Levels of epinephrine similar to those in the blood of stressed individuals greatly inhibited the motility and migration of human keratinocytes (isolated from the foreskin of newborn babies) and of mouse keratinocytes. It also inhibited the repair of scratch wounds made in monolayers of keratinocytes growing on dishes. Treatment of the cultures with a β2AR antagonist (a chemical that prevents epinephrine activating the β2AR) reversed the effects of epinephrine. In addition, the migration of mouse keratinocytes that had been genetically altered so that they did not express β2AR was not inhibited by epinephrine. Next, the researchers investigated the healing of burn wounds made in small pieces of human skin growing in dishes (ex vivo experiments). Burn injuries rapidly increased the amount of epinephrine in these tissue explants, they report, and treatment of the explants with a βAR antagonist (an inhibitor of all types of βARs) greatly increased wound re-epithelialization. Finally, the researchers report that the re-epithelialization of burn wounds in living mice was improved when the mice were treated with a β2AR antagonist.

**What Do These Findings Mean?:**

These findings reveal a second pathway by which stress can impair wound healing. They show that stress-induced increases in systemic and local epinephrine activate β2ARs on keratinocytes and that this activation inhibits keratinocyte motility and wound re-epithelialization. Although results obtained in animals do not always reflect what happens in people, the finding that the treatment of mice with β2AR antagonists improves the rate of burn wound re-epithelialization, suggests that beta blockers—drugs that inhibit all βARs and that are widely used to treat high blood pressure and to prevent heart disease—or specific β2AR antagonists might provide a new therapeutic approach to the treatment of burns and, perhaps, chronic nonhealing wounds.

**Additional Information.:**

Please access these Web sites via the online version of this summary at http://dx.doi.org/10.1371/journal.pmed.1000012.

- Wikipedia has pages on wound healing, burn injuries, and epinephrine (Note: Wikipedia is a free online encyclopedia that anyone can edit; available in several languages)
- The MedlinePlus Encyclopedia has a page on burns (in English and Spanish)
- MedlinePlus provides links to other information on burns (in English and Spanish)

## Introduction

Major burn injury, characterized by burns of greater than 20% of body surface area, is associated with significant multisystem dysfunction [1]. The absence of a protective epithelium in the burn wound results in the comorbidities of fluid and electrolyte loss and wound infection. Despite recent therapeutic advances, the mortality rate from major burns remains high, with a resultant pressing need for the development of economical and widely available therapeutic approaches to enhance the rate of wound re-epithelialization and restoration of the protective epithelial barrier.

Major burns also result in the massive systemic release of the physiological “stress hormones” cortisol and catecholamines from the hypothalamic-pituitary-adrenal (HPA) and the sympathetic adrenomedullary axes (reviewed in [1]). Systemic levels of these mediators are acutely increased and remain elevated for days or weeks after the initial burn trauma [2,3], creating both an acute and chronic stress state in the affected individual. Of the myriad deleterious effects ascribed to stress, one area that has received recent attention is how stress impacts upon wound healing. Multiple forms of stress, as generated, for example, either by physical restraint in mice [4], or in humans, by being a long-term caregiver to demented family members, can delay the healing of an excisional skin wound [5]. The skin's epithelial layer is particularly vulnerable to stress, with stress-induced delays in wound re-epithelialization and ability to repair the skin permeability barrier [6,7]. In seeking to understand the pathobiology of these stress-related impairments of healing, most investigators have focused on the role of cortisol, and indeed, administration of corticosteroids can mimic, while inhibition of glucocorticoid synthesis can abrogate or reverse, the stress-induced skin abnormalities in healing or barrier function [4,7]. In this study, we investigated the alternate hypothesis that the release of stress-related catecholamines, in particular epinephrine, provides a mechanistic link between stress and impaired healing. Because it is paradigmatic of both acute and chronic stress states with long term persistence of elevated levels of circulating epinephrine, the burn wound model served as the basis for the investigation.

Considerable indirect evidence suggests that epinephrine could potentially impair wound healing. Keratinocytes express the β2-adrenergic receptor (β2AR), and its activation by the synthetic catecholamine isoproterenol decreases human skin-derived keratinocyte migratory speed, delays healing of an in vitro scratch wound in confluent keratinocyte monolayers, impairs healing of an excisional human skin wound in organ culture, and delays healing of acute surgical wounds in vivo [8–12]. These effects are reversed by β-adrenergic receptor (βAR) antagonists implicating a βAR-mediated mechanism [13]. Activation of the β2AR in keratinocytes recruits the phosphatase PP2A and ERK to the receptor and results in a decrease in ERK phosphorylation that is required for migration [12]. Keratinocyte migration is required for wound re-epithelialization, and for the final and defining step of wound closure. Thus, if high levels of systemic epinephrine in the chronically stressed patient activate keratinocyte β2AR, impairment of wound re-epithelialization could be predicted to ensue. To test this hypothesis, we used three different experimental models. We first evaluated cell migration in primary cell cultures of human or murine keratinocytes treated with either epinephrine or β2AR- specific agonists or antagonists. Second, we employed an ex vivo human skin explant system to study re-epithelialization of burn wounds. Finally, we investigated the utility of β2AR antagonist treatment for enhancing the rate of burn wound closure in vivo in a mouse model.

## Methods

### Cell Culture

Primary keratinocytes cultures were isolated from neonatal human foreskins as described [14]. Cells were grown in keratinocyte serum-free growth media (KGM, Cascade Biologics) in humidified chambers at 37 °C and 5% CO_2_.

### Cell Migration Studies

Keratinocytes were plated at a density of 50 cells/mm^2^ onto collagen-coated coverslips (60 μg/ml) (Sigma) for 2 h at 37 °C. To simulate the high epinephrine environment seen in burn patients [2], culture medium was supplemented with epinephrine (10 nM) (Sigma). To test for receptor-mediated effects of epinephrine, in some experiments the β1/2 AR antagonist timolol (10 μM) (Sigma) was included in the incubation medium. To demonstrate receptor subtype specificity, some keratinocyte migration studies were performed with cells that had been incubated with β2AR-specific agonist procaterol (10 nM) (Tocris) or β2AR-specific antagonist ICI118,551 hydrochloride (20 μM) (Tocris). To determine the contribution of the phosphatidylinositol 3-kinase (PI3K)/AKT pathway to keratinocyte migration, some assays included pretreatment of cells with the PI3K inhibitor, LY294002 (50 μM, Cell Signaling Technology). Cell migration was monitored microscopically as previously described [11]. Briefly, cells were plated onto collagen-coated glass coverslips at a density of 50 cells/mm^2^ for 2 h at 37 °C. The coverslips were then placed into a migration chamber to monitor individual cell migration over a 1-h period at 37 °C. The migration chamber was placed on an inverted Nikon Diaphot microscope. Time lapse images of the cell migratory paths were digitally captured by Openlab imaging software (Improvision) on a Power Macintosh 8500/120 every 10 min for a 1-h period and imported to a recompiled version of NIH Image 1.60. After each cell's center of mass was tracked, the data were automatically exported to FileMaker Pro 3.0. Data on migration speed for each slide were averaged across cells measured, so that the data analyzed are statistically independent. These data were analyzed in the R statistical package [15] using one-way ANOVA and Tukey's honestly significant difference (HSD) multiple comparisons method. p-Values less than 0.05 were taken to be statistically significant.

### In Vitro Scratch Wounding

Keratinocyte cultures were grown to confluence on 35 mm^2^ plastic tissue culture dishes and wounded with a sterile pipette tip as previously described [11]. A designated area of the wound was photographed from the time of wounding (time 0) until wound closure and the percentage of wound closure was calculated using Openlab imaging software. To prevent cell proliferation from confounding the results, wounded cultures were pre-incubated with 10 μg/ml mitomycin C for 1 h at 37 °C. Percent reduction in wound size was calculated for 12 dishes each treated with epinephrine, epinephrine + timolol, and untreated, at 16 and 24 h in each of three conditions. The data were analyzed as a repeated measures study using the R procedure lme [15] with dish as a random effect and treatment and time as fixed effects.

### Ex Vivo Burn Wounds

We adapted an ex vivo human burn wound healing model developed by Emanuelsson and Kratz [16]. Normal human skin was obtained from surgical procedures for cosmetic breast reduction or abdominoplasty under an approved exemption granted by the Internal Review Board at University of California, Davis. Linear brass rods were heated to 200 °C and placed in contact with the skin for 1 s to create a well-demarcated linear burn. The burned skin samples (2 × 10 mm sections) were immediately transferred to a 12-well dish and submerged in 2 ml of DMEM (Dulbecco's modified Eagle's medium, Invitrogen) containing 10% fetal bovine serum (Tissue Culture Biologicals) and antibiotics, and incubated at 37 °C in a humidified atmosphere of 5% CO_2_ with daily changes of medium.

At the specific time points, the excised burn wounds were fixed in 10% formalin, and embedded in paraffin. Hematoxylin and eosin stained sections (5 μm) were imaged for wound re-epithelialization, as previously described [12,13]. Specimens that were damaged in the histologic process or otherwise not interpretable were excluded from the study. Because hair follicles can serve as an alternate source of re-epithelialization, sections that included hair follicles in the vicinity of the burn wound were also excluded. The percentage of re-epithelialization was calculated by measuring the distance covered by new epithelium divided by the distance between the original wound margins. The new epithelium was clearly differentiated from the nonwounded epithelium at the wound margin by the presence of a fully stratified epithelium and fully formed stratum corneum in the latter. To eliminate observer bias, the percentage of re-epithelialization was calculated from randomly numbered images of the wounds taken by another observer who was blinded to the treatment modality. Although the tissue samples came from only two subjects, a repeated measures analysis using the R procedure lme showed no significant within-subject correlations. Each tissue sample was measured three times and the average of the three was used for analysis. Since each tissue sample was used only at one time and with one treatment, and since there is no significant within-subject correlation, it is appropriate to consider them statistically independent (results when analyzed as a repeated measures model with subject as the random factor were essentially identical to the fixed effects version omitting subject). The data were analyzed using two-way ANOVA with interaction and Tukey's HSD test for multiple comparisons. *p*-Values less than 0.05 were taken to be statistically significant.

### Western Blotting

Ex vivo human skin tissue was burn wounded and then a rectangular 5 × 10-mm area encompassing the wound and the adjacent unwounded tissue was excised immediately after wounding (time 0), at 2 h, and 24 h. An unburned section of tissue was used as control. The tissue was flash frozen in liquid nitrogen, pulverized into a fine powder, and solubilized in RIPA buffer (Sigma) that was augmented with 0.1.% Triton-X and Complete Protease Inhibitor Cocktail (Roche). The tissue solution was then sonicated thrice for 15 s on ice, centrifuged at 14,000 rpm for 20 min at 4 °C, and the supernatant was collected. Protein concentrations were determined using the Bradford protein assay (Bio-Rad Laboratories), and samples were stored at −80 °C. For immunoblotting of cell lysates, treated keratinocyte cultures were washed with PBS containing phosphate inhibitors (50 mM NaF, 1 mM Na_3_VO_4_) and then lysed with lysis buffer (0.5% Triton X in 1× PBS, 50 mM NaF, 1× PBS, 1 mM Na_3_VO_4_, 1× inhibitor cocktail, 0.2 mg/ml PMSF) for 30 min in ice with vortexing. The lysates were centrifugated at 14,000*g* for 20 min at 4 °C. Samples with equal amounts of protein were loaded into a 10% polyacrylamide gel, and SDS-PAGE was run at 200 mV, transferred onto a PVDF or nitrocellulose membrane and incubated overnight at 4 °C with antibodies against either phenylethanolamine-N-methyltransferase (PNMT, Santa Cruz Biotechnology) at a dilution of 1:200, or a 1:1,000 dilution of anti-p-AKT (Cell Signaling Technology), or 1:750 dilution of anti-total AKT (Cell Signaling Technology). The immunoreactive proteins were detected using anti-rabbit horseradish peroxidase-linked anti-IgG (Santa Cruz Biotechnology or Cell Signaling Technology) visualized with ECL western blotting detection reagents (Amersham Life Science). Images were obtained by scanning the films on a UMAX S-6E scanner. Densitometric analysis of scanned images was performed using the NIH public domain ImageJ program, yielding an optical density measurement for each analyte on each blot. Data were analyzed statistically using Student's *t*-test comparing treatment and control in each case. Significance was taken as *p*-values less than 0.05.

### Immunohistochemistry

Human burn skin wounds were flash frozen in liquid nitrogen and 5-μm sections were cut and immediately fixed in ice-cold methanol for 10 min. After blocking endogenous peroxidases with 3% hydrogen peroxide in methanol for 5 min, sections were incubated overnight at 4 °C with rabbit primary antibodies against PNMT (Santa Cruz Biotechnology) diluted 1:20. Visualization of immunoreactants was achieved with biotinylated donkey anti-rabbit secondary antibodies (Santa Cruz Biotechnology), the Vectastain Elite ABC kit (Vector Laboratories), and the Vector DAB kit (Vector Laboratories). Negative controls were incubated in rabbit IgG instead of the primary antibody and all subsequent steps were identical. To visualize the actin cytoskeleton and focal adhesions in cultured keratinocytes, after drug treatments paralleling those of the cell migration assays, cells plated onto collagen-coated coverslips were washed with PBS, fixed with 4% formaldehyde (Fisher Scientific) in PBS, and permeabilized with 0.1% Triton-X/PBS with intermediate PBS washing. Cells were blocked with 5% BSA/PBS 1 h at room temperature and then incubated with monoclonal anti-vinculin antibody (Sigma) at 1:300 dilution in 5% BSA/PBS overnight at 4 °C. On the following day, the cells were washed and incubated with AF594 goat anti-mouse IgG (Invitrogen) at 1:1,000 dilution, Alexa488 Phallodin (Molecular Probes) for actin at 1:200 dilution in 5% BSA/PBS for 2 h at room temperature. The coverslips were washed and mounted onto microscope slides with ProLong Gold antifade reagent with DAPI mounting medium (Molecular Probes). Fluorescent images were captured on a Nikon TE-2000 microscope using Openlab imaging software.

### Reverse Enzyme Immunoassay for Epinephrine

For ex vivo tissue epinephrine measurements, supernates of flash frozen, pulverized burn tissue were processed and stored as for western blotting (above). For plasma measurements in burn wounded mice, blood was removed by cardiac puncture with heparin pretreated syringes and centrifuged. The supernatant was collected, acidified to 0.01 M HCl, and stored at −80 °C. Extracts from tissue or plasma were tested in triplicate with an epinephrine enzyme immunoassay (BIOSOURCE) according to the manufacturer's instructions, as we have previously reported [13]. The level of epinephrine in tissue extracts was expressed as pg of epinephrine/mg of protein.

### In Vivo Burn Wounds

The murine burn wound protocol [17] approved by the Animal Use Committee, University of California, Davis, California used female FVB/NJ mice (Jackson Labs, stock number 001800), anesthetized throughout the procedure with isoflurane. The hair on the back and the sides were shaved and a small incision was made was made in the side for the subcutaneous implantation of continuous infusion osmotic pumps (Alzet Corporation, stock number 2002). The pumps delivered either saline (control), 3 mg/kg/d salbutamol (β2AR specific agonist), or 0.7 mg/kg/d ICI-118,551 (β2AR specific antagonist). A flame-resistant template of appropriate size to produce a 20% body surface area burn was placed over the shaved surface of the animals, and an ethanol flame burn was delivered to the area. The procedure produced a full-thickness burn with discrete borders measuring 1.8 cm by 4.5 cm. Immediately after the burn, analgesia was administered (buprenorphine 0.5 mg/kg in 0.4 ml of sterile saline, IP) and an additional 0.6 ml sterile saline subcutaneously for postburn fluid resuscitation. Buprenorphine IP was administered every 8 h for pain management for the first three recovery days. Sham control animals were anesthetized, shaved, and had 1 ml of 70% ethanol pipetted onto their backs, but were not burned. These animals were injected with buprenorphine in saline while anesthetized and received buprenorphine IP each day for the first 3 d following the sham procedure as did the test animals.

At specific days postburn wounding, mice were killed by cardiac puncture. Burn wounds were excised with a margin of unburned skin and fixed overnight in 10% phosphate buffered formalin and embedded in paraffin. Hematoxylin and eosin stained sections were scored for re-epithelialization using light microscopy by an examiner blinded to the treatment modalities. Since each mouse had only one treatment and was measured only at one time, it is appropriate to consider them statistically independent. The data were analyzed using two-way ANOVA with interaction and Tukey's HSD test for multiple comparisons. *p*-Values less than 0.05 were taken to be statistically significant.

## Results

**Figure 1:**
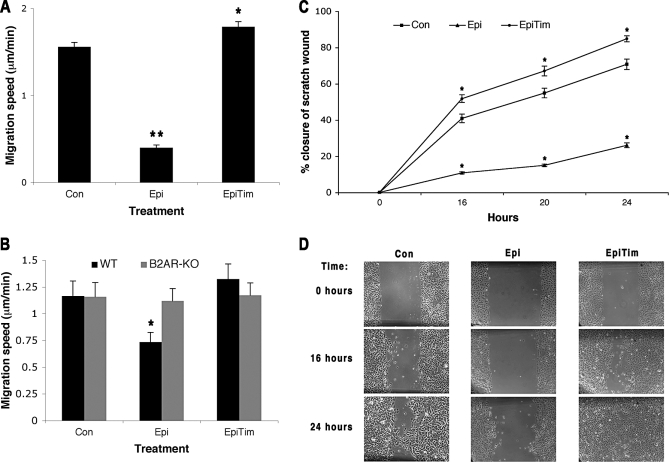
Epinephrine Decreases Keratinocyte Migratory Speed and In Vitro Scratch Wound Healing by Activation of the β2AR (A) Neonatal human keratinocytes (NHK) migratory speeds were measured for 1 h after treatment with epinephrine (10 nM), epinephrine (10 nM) and timolol (10 μM, nonspecific βAR antagonist), or no added drug (control). Error bars represent standard error of the mean, *n =* 80; * p < 0.05; ** p < 0.001. (B) Epidermal keratinocytes were cultured from FVB β2AR knockout mice (β2AR-KO, β2AR −/−) and wild-type controls (WT, β2AR +/+) and assessed for their migratory speeds, as in Figure 1A. Error bars represent standard error of the mean, *n =* 31; * p < 0.01. (C) NHK in vitro scratch wound closure after control, epinephrine, and timolol treatments. Error bars represent standard error of the mean, *n =* 12; * p < 0.05; ** p < 0.001. (D) Representative pictures from the scratch wound assay are shown. Scale bar = 100 μm.

To determine if epinephrine could directly modulate keratinocyte motility, we treated cultured human keratinocytes with epinephrine at levels that are comparable to that found in the circulation of burn-wounded patients (10 nM) [2]. Epinephrine decreases both keratinocyte migratory speed (from 1.587 to 0.388, 95% confidence interval [CI] for the difference using Tukey's HSD 0.891–1.508, *p* = 0.00001) and in vitro scratch wound closure rates (difference in percent reduction from baseline of −30.11% with 95% CI −33.33% to −26.89%, *p <* 0.0001), while the addition of a βAR antagonist, timolol, reverses these effects (Figure 1A, migration speed 1.791, 95% CI for the difference between timolol + epinephrine and epinephrine alone 1.070–1.736, *p =* 0.00001 for cell migration, difference in percent reduction from baseline between timolol+epinephrine and epinephrine alone 34.55% with 95% CI 31.33%–37.77%, *p <* 0.0001 for in vitro scratch wounding) suggesting that the epinephrine effects are mediated through the βAR. The specific receptor dependence of the observed response was confirmed by examining the migratory rates of keratinocytes isolated from the epidermis of mice in which the β2AR had been genetically deleted [18]. Wild-type β2AR expressing keratinocytes are slowed by the inclusion of 10 nM epinephrine (from a speed of 1.165 to 0.735, 95% CI for the difference 0.28–0.58 by Tukeys's HSD, *p =* 0.001 ) while the β2AR −/− keratinocytes show no response in migratory rate to either agonist or antagonist (Figure 1B, all differences less than 0.06 with an overall mean of 1.15, none significant).

**Figure 2:**
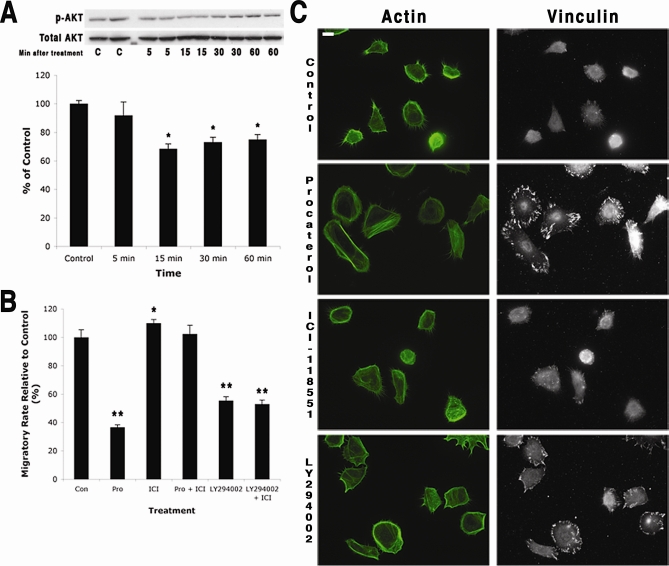
The β2AR Pathway Involves PI3K/AKT in Controlling Keratinocyte Migration (A) (Top) Keratinocytes were treated with procaterol (10 nM, specific β2AR agonist), and the cells were collected at sequential time points and assayed for the expression of phosphorylated AKT (p-AKT) and total AKT. A representative western blot (of two different human keratinocyte strains) is shown. Untreated keratinocytes served as control. (Bottom) p-AKT expression was significantly decreased at 15, 30, and 60 min after procaterol (10 nM) treatment. Error bars represent standard error of the mean, *n =* 4; * p < 0.05. (B) Keratinocyte migration was measured after exposure to either procaterol (10 nM), ICI-118,551 (20 μM, specific β2AR antagonist), procaterol with ICI-118,551, LY294002 (50 μM, PI3K inhibitor), or LY294002 (50 μM) with ICI-118,551 (20 μM). Untreated keratinocytes served as controls. Data were pooled from results from two different human keratinocyte strains. Error bars represent standard error of the mean, *n =* 67–106; *, p < 0.05; **, p < 0.001. (C) Keratinocytes immunostained for the expression of actin and vinculin after they were treated with either procaterol (10 nM), ICI-118,551 (20 μM), or LY294002 (50 μM). Procaterol and LY294002 both induced similar changes in the cytoskeletal organization of vinculin whereas ICI-118,551 did not. Untreated keratinocytes served as controls. Scale bar = 15 μm

β2AR activation has been previously demonstrated to mediate migration in keratinocytes by regulating the promigratory ERK signaling pathway [10–12]. However, recent studies have also implicated the PI3K/AKT pathway as a downstream mediator of β2AR activation in some cell types [19,20]. Since the PI3K/AKT pathway controls actin dynamics and thus cell migration [21,22], we evaluated this mechanistic possibility for β2AR-mediated reduction of locomotory speed in keratinocytes. For these studies, the highly selective and potent β2AR agonist procaterol (*K*_i_ for β2AR 70 nM) [23] and the highly selective β2AR antagonist ICI-118,551 [24] were used. AKT activation was decreased in keratinocytes cultured with the β2AR-selective agonist procaterol (Figure 2A). Inhibiting the AKT signaling pathway by incubating keratinocytes with LY294002, the selective inhibitor for the directly upstream PI3K, [25] resulted in a marked reduction in migration rate (Figure 2B), as did incubation with the β2AR-selective agonist procaterol. The β2AR agonist-mediated decrease in migratory speed is abolished if the cells are co-incubated with the β2AR-specific antagonist, demonstrating dependence of the response on receptor activation. Although the β2AR-selective antagonist alone increased keratinocyte migratory speed (Figure 2B), this increase was abrogated when the PI3K inhibitor LY294002 was included in the culture incubation, indicating that the PI3K/AKT signaling are downstream of β2AR activation. Keratinocytes treated with either the β2AR-selective agonist procaterol or the inhibitor of PI3K LY294002, both demonstrated a cell phenotype associated with stationary cells: increased numbers of large focal adhesions and transcellular stress fibers (Figure 2C). Keratinocytes in control or β2AR antagonist conditions (Figure 2C), demonstrated abundant filopodia and smaller/fewer focal adhesions, characteristic of the migratory epithelial phenotype [22,26,27]. Together these findings suggest that activation of the β2AR in keratinocytes results in decreased signaling through the PI3K/AKT pathway with the outcome of a nonmigratory cell phenotype and decreased locomotory speed. Since the keratinocyte β2AR regulates its motility, it is logical to propose that in a stressed individual, elevated levels of circulating epinephrine could contribute to impaired wound healing by delaying the re-epithelialization process that is wholly dependent on the rate of keratinocyte migration.

**Figure 3:**
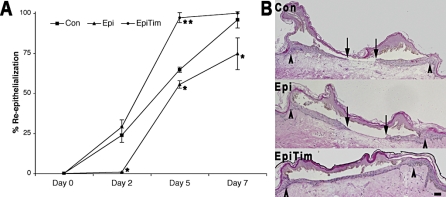
Ex vivo Burn Wound Re-epithelialization Is Inhibited by Epinephrine (A) Ex vivo human skin burn wounds (2 mm wide) were treated with either epinephrine (10 nM) (Epi), or with the combination of epinephrine (10 nM) and timolol (10 μM) (EpiTim). Control (Con) treatment consisted of DMEM with 10% FBS. Re-epithelialization was monitored by histology followed over 7 d. Error bars represent standard error of the mean, *n =* 3; * p < 0.05; ** p < 0.01. (B) Representative day 7 burn wound histological cross-sections are shown. Arrows represent the wound edge and the arrowheads represent the edge of re-epithelialization. Scale bar = 100 μm.

To test this hypothesis, we used a human skin burn wound model. Burn wounds generate a stressed condition characterized by sustained, high-circulating levels of epinephrine, as much as 10-fold increased over the nonstressed state [1,2]. To emulate the clinical high-epinephrine stress environment in burn wounds, excised normal human skin was burn-wounded [16] and incubated in culture medium that contained either epinephrine (equivalent to plasma levels in the burn patient, 10 nM) or epinephrine along with the βAR antagonist timolol. Re-epithelialization of the burn wounds was assessed by histomorphometric analysis of excised wound tissue over a 7-d interval. By day 2, wounds in the elevated epinephrine culture environment display delayed re-epithelialization (Figure 3A, 95% CI for the difference 10.2%–35.9% by Tukey's HSD, *p =* 0.0036). Inclusion of the βAR antagonist timolol into the treatment medium blocks this delay (Figure 3A, 95% CI for the difference 15.4%–41.0% by Tukey's HSD, *p =* 0.0012). Differences in re-epithelialization are also evident at days 5 and 7, and almost all wounds that were treated with the epinephrine and timolol combination are completely re-epithelialized by day 5 (Figure 3A and 3B), while the epithelialization of the epinephrine treated wounds lags behind. These findings indicate that elevated levels of epinephrine inhibit re-epithelialization of burn-wounded human skin and, importantly, that blockade of the βAR presents an apt therapeutic target for reversing the epinephrine-mediated impairment in wound re-epithelialization.

**Figure 4:**
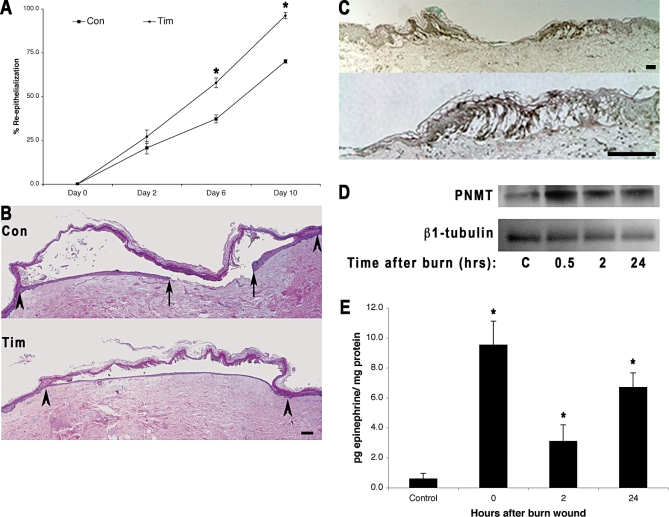
Human Skin Generates Local Epinephrine Ex Vivo in Response to Burn Injury (A) Ex vivo human burn wounds (2.5 mm wide) in one piece of donor skin were divided into three groups that received either no treatment (CON) or timolol (10 μM ) (TIM). It should be noted that these burn wounds are larger in width than those in Figure 2, but the relative rate at which the timolol treated group re-epithelializes is consistent at approximately twice the speed of the control by day 5 (Figure 2A) or day 6 (Figure 3A). Error bars represent standard error of the mean, *n =* 5; * p < 0.01. (B) Representative day 10 burn wound histological sections are shown. Arrows represent the wound edge and the arrowheads represent the edge of re-epithelialization. Scale bar = 100 μm. (C) Human skin burn wounds were examined for the expression of PNMT by immunohistochemistry at day 1 after burn wounding. Increased expression of PNMT was found in the epidermis immediately adjacent to the burn wound margins. Scale bar = 100 μm. (D) Western blot for PNMT expression in lysates of peri-wound skin. Lane 1, unburned tissue control; lane 2, 30 min postburn; lane 3, 2 h postburn; and lane 4, 24 h postburn. (E) Epinephrine levels were measured in unburned skin (control), immediately (time 0), 2 h, and 24 h after burn wounding. Error bars represent standard error of the mean, *n =* 3, * p < 0.001.

It should be noted that when the burn-wounded skin was incubated with the βAR antagonist timolol, wound re-epithelialization is accelerated (Figure 4A and 4B). Since this process occurs in the absence of exogenously added catecholamines, this finding prompts the question of whether the antagonist could be blocking an endogenous agonist that is locally generated by the wounded skin. Previous work has demonstrated that keratinocytes express PNMT, the enzyme necessary for the conversion of norepinephrine to epinephrine, and epinephrine has been detected in keratinocyte lysates, suggesting that keratinocytes can synthesize epinephrine [13,28,29]. We therefore investigated whether burn wounding the epidermis can up-regulate this terminal enzyme of the epithelial catecholaminergic pathway, resulting in autocrine stimulation of the β2AR. Indeed, expression of PNMT increases in the epidermis immediately adjacent to the burn wound (Figure 4C) relative to the limited expression in the more distal and uninjured epidermis. PNMT expression is also limited in the dermis. PNMT protein expression increases in the skin as early as 0.5 h after burn wounding and remains elevated for at least 24 h (Figure 4D). Levels of epinephrine are also elevated in the burned wounded tissue, with an immediate 15-fold elevation over preburn levels, and sustained elevation through 24 h (Figure 4E). These results in the ex vivo burn wound, where there is an absence of a systemic circulation and exogenously supplied catecholamines, suggest a likely scenario where burn wounding of skin induces local epidermal generation of epinephrine by up-regulation of the enzyme required for its synthesis in the peri-wound epidermis. Taken together, these data demonstrate that β2AR blockade, even in the absence of systemic elevations of epinephrine, can improve burn wound re-epithelialization and suggests the utility of beta blockers as a pharmacologic approach to improve burn wound healing in vivo.

**Figure 5:**
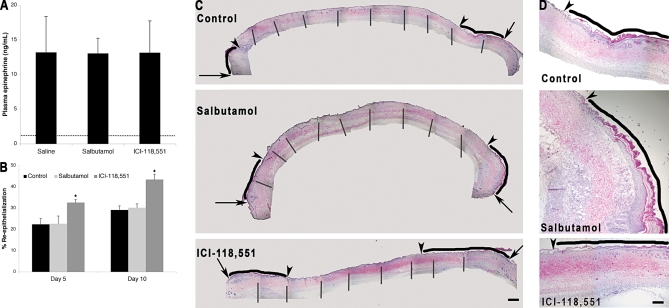
In Vivo Burn Wound Re-epithelialization Is Accelerated by β2AR Blockade Mice were subjected to a 20% body surface area full thickness burn and were treated using continuous infusion pumps with either saline (control), 3 kg/mg/d salbutamol (β2AR specific agonist), or 0.7 kg/mg/d ICI-118,551 (β2AR specific antagonist). (A) Plasma epinephrine levels were measured in each animal at day 10 postburn wounding. The mean preburn level of epinephrine (dashed line) was determined from plasma samples taken immediately following burn wounding. Error bars represent standard error of the mean, *n =* 5. (B) Wound re-epithelialization was measured by image analysis of histologic sections of the wounds excised on either day 5 or day 10 postburn wounding. Error bars represent standard error of the mean, *n =* 5, * *p <* 0.001. (C) Representative histological sections from day 10 burn wounds are shown. Arrows represent the wound edge and the arrowheads represent the edge of re-epithelialization. The blacks lines indicate the zone of re-epithelialization. The overall wound images are a montage compiled from multiple images as indicated by vertical bars. Scale bar = 1 mm. (D) Magnified images showing the re-epithelializing edge from the right side of the pictures in (C). Arrowheads represent the edge of re-epithelialization and the black lines indicate the zone of re-epithelialization. Scale bar = 4 mm.

To directly test the hypothesis that β2AR blockade could improve burn wound re-epithelialization in vivo, a murine burn wound model was employed. Female FVB/NJ mice were subjected to a 20% body surface area full-thickness burn [17], and implanted osmotic pumps delivered either saline, salbutamol (β2AR agonist), or ICI-118,551 (β2AR antagonist) as treatments over a 10-d course. Wound re-epithelialization was determined by histomorphometric analysis of the wounds excised on either day 5 or 10. Plasma epinephrine levels remained 10-fold elevated over preburn levels in all treatment groups over this time period, confirming the high-epinephrine stress state that accompanies burn wounds of this magnitude (Figure 5A). The β2AR antagonist treated groups showed a significant increase in wound re-epithelialization at both days 5 and 10. (The mean re-epithelialization was 26.7% for the control, 26.1% for the agonist, and 38.2% for the antagonist. The 95% CI for the main effect difference between control and antagonist using Tukey's HSD is 8.3%–16.7%, *p <* 0.0000001, the interaction between day and treatments was not significant.) On the other hand, there is no significant difference in re-epithelialization between the saline-treated and the β2AR agonist-treated groups (Figure 5B–5D), likely because the high levels of circulating epinephrine in both of these groups maximally delayed wound re-epithelialization, which is then not further diminished by the treatment with the β2AR agonist. Interestingly, we did not note any significant histologically observable alteration in the dermal wound granulation tissue for any of the treatments (unpublished data). The animal data demonstrate that systemic treatment with a β2AR antagonist accelerates wound re-epithelialization in the setting of a high epinephrine stress environment, such as accompanying a major burn wound.

## Discussion

Taken together, these studies demonstrate how stress, with its associated elevation of the stress hormone epinephrine, can impair cutaneous wound healing: by activation of the epidermal keratinocyte β2AR, blunting of promigratory signaling pathways, stabilizing a stationary cell morphologic phenotype, and subsequently diminishing migratory speed required for efficient wound re-epithelialization. This work also expands the developing understanding of how stress can modulate wound healing, which until now has focused primarily on the hypothalamic-pituitary-adrenal (HPA) and glucocorticoid-mediated pathway. The results presented here demonstrate that the neuroendocrine stress response, with the emblematic increase in epinephrine, can also directly affect the wound repair process. Both genetic and pharmacologic approaches demonstrate the dependence of the epinephrine-mediated impairment on an intact keratinocyte β2AR, and implicate this receptor in the impairment of healing in the burn-wound stress model used here. While some of the pathways for glucocorticoid-induced impairment of healing have been elucidated [4,7], the glucocorticoid and catecholamine pathways may crosstalk to impact healing. Here we note a burn-induced rapid rise in epidermal PNMT in the immediate burn wound vicinity. Levels of this epinephrine synthesizing enzyme have been reported to be elevated by stress in multiple tissue by both glucocorticoid-dependent and -independent mechanisms [30]. The PNMT promoter has a glucocorticoid response element [31], and elevations in circulating cortisol induce rapid increases in the PNMT transcripts and protein expression [32]. Interestingly, epidermal keratinocytes have recently been found to have glucocorticoid synthesizing ability, and this synthesis is increased in wounding [33,34]. Thus, a local hormonal signaling pathway can be envisioned, where wounding induces epidermal glucocorticoid synthesis, which can drive the observed up-regulation of PNMT, and where temporal imbalances in the pathway could result in sustained elevation of both local glucocorticoids and catecholamines, with resultant impairment of healing.

Activation of the β2AR in some cell types, including cardiac myocytes, corneal and breast epithelial cells, and skin keratinocytes, decreases phosphorylation of the promigratory ERK signaling pathway [10,11,35–37], thus contributing to a decrease in migratory speed, and impaired wound closure seen in epithelia exposed to βAR agonists [12,36]. However, other signaling pathways coregulate cell migration—among them, the PI3K/AKT pathway [19,20]. Through not yet fully identified downstream targets, activation of this pathway results in reorganization of the actin cytoskeleton and promotes cell migration [21,22]. Here we find that activation of β2AR in keratinocytes attenuates promigratory PI3K/AKT signaling. Directly inhibiting this pathway by incubation of keratinocytes with an inhibitor of PI3K results in decreased cell motility, and induces a stationary cell phenotype, mimicking the findings observed when cells are incubated with specific β2AR agonists. Thus, in addition to the previously demonstrated β2AR modulation of the ERK signaling pathway, this work uncovers a new mechanistic signaling link between the β2AR and the PI3K/AKT pathway in modulating keratinocyte migration. These two pathways converge to result in decreased keratinocyte migration and subsequent impaired wound re-epithelialization.

Wound healing rates decrease with age (reviewed by [38,39]) and neonatal human derived keratinocytes have a more robust migratory response to promigratory factors as compared to adult human keratinocytes [40]. It should be noted that our experiments investigate both neonatal human keratinocytes in the in vitro studies and adult human keratinocytes in the ex vivo burn wounds, and we find similar results in both sets of experiments. Therefore, a strength in this study is that the results are applicable to re-epithelialization of wounds in the young and the aged.

These findings do not exclude a role for epinephrine in modulating the inflammatory, vascular, and dermal fibroblast components of wound repair. Sustained elevations in the level of plasma epinephrine are implicated in stress-induced immunosuppression that accompanies depression [41], and have been implicated in tumor angiogenesis contributing to tumor growth [42]. Apparently some basal catecholamine level is required for wound healing since other studies have shown that total norepinephrine depletion leads to impairment in healing of murine acute surgical wounds [43,44]. Additionally, wound contraction mediated by the highly contractile myofibroblasts that populate wound granulation tissue serves an important role in murine and loose-skinned rodent—but notably not in human—wound healing [45]. Although it has previously been shown that elevated epinephrine inhibits fibroblast proliferation [46], the role of epinephrine on granulation tissue formation remains unknown and will need to be addressed in future investigations. Interestingly, it has been shown that alpha, and not beta, adrenergic receptor blockade can improve stress-induced impairments in wound contraction [47], and likely this is mediated by dermal myofibroblasts. Since wound contraction is not a prominent component of human wound healing, the current study focuses on wound re-epithelialization. No matter how well organized and complete the repair of the dermal or vascular architecture of a wound may be, without the final step of re-epithelialization and its accompanying restoration of the skin barrier, wound healing cannot be considered to be complete. Thus, the current work focuses on the role of stress-induced elevations in epinephrine in this critical step of healing.

Lastly, these results suggest a new therapeutic approach to treating burn and perhaps chronic, nonhealing wounds: beta blockade. Beta blockade is already in use in some patients with severe burns to prevent postburn catecholamine-mediated muscle wasting [1,48]. Because beta blockers have a long history of clinical use, a well-documented safety profile, are inexpensive and widely available, they present a therapeutic target that could have a streamlined translational path. Currently used beta blockers have nonspecific affinity to the βAR subtypes, but the findings presented here demonstrate strong support for the development of specific β2AR antagonists to pharmacologically enhance wound re-epithelialization. Therapeutic interventions targeting the neuroendocrine axis, catecholamine synthesis, and receptor activation thus present novel strategies for addressing stress-related wound impairment.

## Abbreviations

βAR: β-adrenergic receptor
β2AR: β2-adrenergic receptor
CI: confidence interval
HSD: honestly significant difference
PI3K: phosphatidylinositol 3-kinase
PNMT: phenylethanolamine-N-methyltransferase
